# Assessment of Gradient-Based Algorithm for Surface Determination in Multi-Material Gap Measurements by X ray Computed Tomography

**DOI:** 10.3390/ma13245650

**Published:** 2020-12-11

**Authors:** Roberto Jiménez-Pacheco, Sinué Ontiveros, José A. Yagüe-Fabra, Filippo Zanini, Simone Carmignato, José Antonio Albajez

**Affiliations:** 1 Centro Universitario de la Defensa, A.G.M. Carretera Huesca s/n, 50090 Zaragoza, Spain; rjimenez@unizar.es; 2 Department of Industrial Engineering, Autonomous University of Baja California, Mexicali 21100, Mexico; sinue.ontiveros@uabc.edu.mx; 3 I3A, Universidad de Zaragoza, María de Luna 3, 50018 Zaragoza, Spain; jalbajez@unizar.es; 4 Department of Management and Engineering (DTG), University of Padova, Stradella San Nicola 3, 36100 Vicenza, Italy; filippo.zanini@unipd.it (F.Z.); simone.carmignato@unipd.it (S.C.)

**Keywords:** computed tomography, multi-material measurements, gap measurements, surface determination algorithm, gradient-based algorithm

## Abstract

X-ray computed tomography is one of the most promising measurement techniques for the dimensional evaluation of industrial components. However, the inherent complexity of this technology also involves important challenges. One of them is to develop surface determination algorithms capable of providing measurement results with better accuracy in any situation—for example, for single and multi-material parts, inner and outer geometries, with and without image artefacts, etc.—and reducing user influence. The surface determination is particularly complex in the case of multi-material parts, especially when they are separated by small air gaps. In previous works, two gradient-based algorithms were presented, that showed less measurement variability throughout the whole part, and reduced the computational cost and operator influence compared to threshold-based algorithms. This work focuses on the evaluation of the performance of these algorithms when used in a scenario so complex that parts of it are made of one or more materials (metal–metal and polymer–metal) with gaps inside. For this purpose, a set of multi-material reference standards is used. The presented gradient-based algorithms show measurement errors comparable to commercial threshold-based algorithms, but with the capability of obtaining accurate measurements in smaller gaps, apart from reducing the user influence on the measurement process.

## 1. Introduction

X-ray computed tomography (CT) is a non-destructive testing (NDT) technique increasingly used for the dimensional evaluation of industrial components, mainly due to its ability to acquire, in combination with proper post-processing, geometrical information on both internal and external geometries simultaneously in a single scan and in a non-destructive way. In industrial X-ray CT metrology, the absorption mode was typically used. CT offers possibilities in the metrology field that are difficult to achieve by other measurement methods, such as the simultaneous measurement of assembled parts, internal geometries or the metrological analysis of porosity or fibres. However, the inherent complexity of this technology also involves important challenges that the scientific community is trying to solve with the numerous studies carried out in recent years. One of these challenges in industrial X-ray CT metrology is the surface determination of the studied part [1]. Since this surface determination consists of defining the contour of studied objects, this process is critical in dimensional and geometrical verification.

One of the most complex conditions that surface determination algorithms have to face is the analysis of multi-material parts. According to [2], two multi-material scenarios can occur in measurement tasks. In the first scenario, parts made of different materials form an interface, either because they are assembled together or because a part of one material is enclosed within another base material. In the second one, there is a gap between the surfaces of the different material parts. Furthermore, when different materials have a very different absorption coefficient, such as between polymers and metals, the difficulties of making a proper surface determination increase.

The conventional approach to surface determination in metrological applications is based on the principle of similarity, with threshold-based algorithms being the most widely used. In this type of algorithm, a certain range of grey values is used as a reference of similarity, considering as part of the same material all those voxels of the reconstructed 3D volume whose value is in this range. In this way, a segmentation of the 3D volume is generated according to the absorption that it presented during the scanning, since this parameter is the one that shows the grey level. To determine the ranges of grey value that distinguish a material from air or another material, a threshold value that differentiates them is set. The surfaces that enclosed the segmented materials are determined using this threshold value. The first surface determination algorithms developed in industrial metrology applications used static thresholds, the most commonly used being ISO 50% [3], where the threshold is established according to the histogram of grey values of the whole part, choosing the average value between the air or background peaks and the peak of the part material. However, it has been shown that the use of this algorithm, even on single-material parts, often results in surface displacements [4]. For this reason, algorithms based on a static threshold have evolved into methods based on a dynamic threshold, also called local threshold. These algorithms start from an initial segmentation, carried out for example with static threshold techniques. Then, a local adjustment of the threshold is carried out based on the voxel values of the area in which the surface is to be determined. In [3], an experimental evaluation of the influence of a surface determination process on multi-material measurements using functions available in a commercial CT software (Volume Graphics VGStudio MAX) is presented; the results show that the local-adaptive threshold method presents the lower measurement errors. In addition, such work determines that in the case of a high level of noise in the CT volume data, specific pre-processing steps may be necessary. The same conclusion is reported in [5], where it is shown that applying local-adaptive threshold methods can improve the surface determination procedure.

Although local-threshold techniques are able to offer good results in many metrological applications, when measuring multi-material parts whose materials have very different absorption coefficients, it is difficult to find the right scanning parameters for all of them, resulting in some cases in poor quality images. The same issue can also occur for complex scanning geometries such as small gaps. In these situations, an alternative is the use of multi-energy scans, using a specific energy for each material. In [6] a multi-material gap measurement using dual-energy was performed. Although the results are improved in comparison to single source X-ray CT systems, it needs two complete projection sets for each object to be scanned, and this increases the economic cost of the process.

To cover these weaknesses, several segmentation algorithms have appeared in recent years as an alternative to threshold-based surface determination techniques. These algorithms have their strength in the better identification of the different materials, but obviously also end up determining the surface that limits them. In [7], a segmentation algorithm based on the combination of two different techniques is shown. First of all, a technique adapted from the well known method of regional growth is applied to distinguish the different materials and most of their volume. After applying this first phase, certain voxels remain unclassified in any of the materials, most of them corresponding to the edges of the parts. These voxels are finally assigned to one material or another using a graph-cut based algorithm. Although no data are provided for their accuracy and precision from a metrological point of view, the authors recognise difficulties in distinguishing materials with a similar absorption coefficient, especially with noisy CT volume data.

Another approach to surface determination that combines different segmentation methods is presented in [8]. This paper presents a surface determination technique that combines a clustering based segmentation method followed by a region-based segmentation method. Firstly, a fuzzy C-means clustering method is used to pre-process the volume and classify the different objects. Then a region growing algorithm was applied to obtain a dimensionally accurate 3D surface model. The dimensional studies show accurate results, but the proposed methodology shows some limitations regarding the manual selection of the parameters for an appropriate surface determination.

An alternative to similarity-based surface determination algorithms are discontinuity-based algorithms. Techniques based on discontinuity have been widely applied in biological [9] and medical applications [10] and in the image and vision computing field [11]. However, in these applications, the requirements for precision and accuracy are not as demanding as in industrial metrology applications.

An example of discontinuity-based algorithms designed for industrial applications is shown in [12]. In this algorithm, the whole volume is segmented using a multilabel graph-cut in order to get an approximate contour of the parts. From this contour, the direction in which the variation of the voxel grey value was greatest was determined by calculating the gradient. The surface of the part is determined by calculating the local maximum by interpolating the gradient in that direction of the maximum gradient. The main disadvantage of this method is that it requires a user to set the number of the materials and their representative CT values. In addition, the article does not present a dimensional analysis.

The University of Zaragoza (Spain) has also developed two surface determination algorithms based on a gradient. One based on the Canny algorithm [13] and another based on the Deriche algorithm [14,15]. These algorithms have been tested using parts with different geometries: round, flat and freeform [16]. They have also been tested using a polymer multi-material standard [17]. These works show that these algorithms are able to offer similar or even better results than conventional threshold-based algorithms in metrological applications. Furthermore, they allow for a significant reduction in user influence on the measurement results. However, the performance in terms of metrological applications of these algorithms has not been tested for multi-material parts when there is a gap between them. Since gradient-based surface determination algorithms detect the surface based on changes in the grey value, the accuracy of the measurement should not be affected by the difference in absorption coefficients of the materials involved as long as the gap is big enough. However, it is well known that the metrological structural resolution (MSR), defined as the smallest structure measurable with maximum permissible error to be specified, is affected by multiple factors [18], including the surface determination algorithm. In particular, it is necessary to evaluate the ability of the gradient-based algorithms to detect the gap when it is too narrow, around one or few times the voxel size. In this case, due to the partial volume effect [19], the grey value of a voxel represents the average absorption of an area of the part where material and air overlap, and if the gap is smaller than the size of a voxel, the air and the two materials will overlap.

In [2], the University of Padova (Italy) and Physikalisch–Technische Bundesanstalt (PTB, Braunschweig, Germany) presented a set of metal and polymer multi-material reference standards in order to evaluate the performance of different algorithms measuring the air gap between two different materials. In the same work, the main four algorithms based on threshold and local threshold provided by the commercial software VGStudio MAX (Volume Graphics, Heidelberg, Germany) were compared.

The aim of the work here presented was to evaluate the performance of the two aforementioned gradient-based surface determination algorithms developed by the University of Zaragoza, when applied to multi-material (metal and polymer) parts with a gap at the interface, in comparison with conventional algorithms. This performance is evaluated in terms of the capability of the algorithm of detecting the edge of the part when the gap becomes narrower than 0.5 mm, and also in terms of accuracy in comparison with that offered by the main algorithms implemented in VGStudio MAX. To carry out this work, the set of multi-material reference standards developed by the University of Padova and PTB were used.

## 2. Materials and Methods

### 2.1. Reference Standard

**Figure 1:**
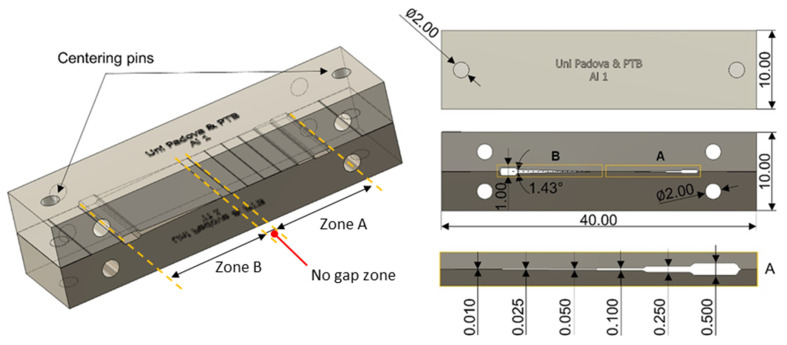
Multi-material gap standard. Dimensions are reported in mm.

In order to evaluate the performance of the analysed algorithms, a set of metal and composite standards designed by the University of Padova and the PTB was used. These standards were presented in [2] and were specifically designed to evaluate the capability of CT systems for measuring gaps in single and multi-material parts. Each of these standards is composed of two parts that can be made of the same or different material. Each one of the two parts has a stepped surface and a tempered plane machined. When the two parts are assembled, stepped air gaps are generated in one part and a wedge air gap in the other. The nominal thickness of the stepped gaps ranges from 500 to 10 µm (zone A in Figure 1) and the nominal wedge gap ranges from 450 to 0 (no gap) micrometres (zone B in Figure 1). A 1 mm step gap is also generated at zone B, just before the wedge zone begins. The design of the reference standard is shown in Figure 1.

Three of the available materials combinations have been selected for this work: Aluminium/Titanium (Al/Ti), Aluminium/Aluminium (Al/Al) and Aluminium/Carbon-fibre reinforced silicon carbide (Al/Cesic^®^). This selection will allow the evaluation of the algorithms in three different configurations, respectively, of materials with very different, identical and similar absorption coefficients. The details of the selected materials can be found in Table 1.

The reference dimensions of the standards were measured using a high-precision tactile micro-coordinate measuring system (CMS) Zeiss F25 with the maximum permissible error (MPE) equal to (0.25 + *L*/666) μm (where *L* is the measuring length in mm) [2].

### 2.2. CT Scanning and Reconstruction

All the reference standards were scanned using a CT system Nikon MCT225 (Nikon Metrology, Inc, Brighton, MI, USA), setting the parameters shown in Table 2. These parameters were set to obtain the best image quality and MSR [18], taking into account the sample geometry, dimensions and materials. The different exposure time and copper filter thickness used for the Al/Ti standard were necessary to cope with the higher X-ray absorption coefficient of titanium with respect to the other materials and to reduce the increasing beam hardening effect occurring in such material configuration. As reported in Table 2, the voxel size achieved to acquire the entire sample in a single scan was equal to 22.1 µm. The temperature during the scans was maintained within the range of 20 ± 0.5 °C.

All the acquired images were reconstructed using the software CT PRO 3D (Nikon Metrology, Inc, Brighton, MI, USA), provided by Nikon Metrology.

### 2.3. Surface Determination Algorithm and Measurement

To carry out the surface determination process, CT reconstructed volumes were processed using two different algorithms developed by the University of Zaragoza and implemented in Matlab. Both are based on gradient determination and they were already implemented in previous works [13,14], leading to better results in terms of precision and accuracy than conventional threshold methods [15].

The first surface determination method is based on the Canny algorithm [13]. This algorithm performs a gradient determination using a Canny-based filter along the three Cartesians directions (X, Y and Z). With these data, a preliminary edge detection was carried out. After that, the surface point was determined with a sub-voxel refinement using the same gradient data. This algorithm was characterized by the low influence on the accuracy of the software operator. This also offers good results in terms of repeatability, especially on flat surfaces. However, it involves an important computational cost.

The second surface determination method is based on Deriche algorithm [14,15]. In this algorithm, the preliminary edge detection is carried out using a faster operator, adapted from the Deriche gradient operator. The sub-voxel resolution refinement is performed considering the local maximum gradient direction. This algorithm reduces the computational cost significantly compared to the previous algorithm [15], which allows to improve the sub-voxel refinement sub-process, achieving a higher accuracy of the algorithm, especially when it is applied to dimensions defined by flat surfaces. In addition, this algorithm enables a further reduction in the influence of the software operator.

In order to compare the performance of the gradient-based algorithms studied in this work with conventional threshold algorithms, the same CT reconstructed volumes were processed also using the commercial analysis and visualization software VGStudio MAX (Volume Graphics, Heidelberg, Germany). In particular, four different surface determination procedures using the algorithms included in VGStudio MAX were implemented in this work: ISO-50% global threshold applied to the complete dataset (ISO), ISO-50% local-adaptive threshold applied to the complete dataset (ISO-adv), ISO-50% with double surface determination using two different global threshold values for the two different materials (ISO-double) and ISO-50% with double surface determination and local-adaptive threshold (ISO-double-adv). The last two algorithms were used as they were proven to be more suited for multi-materials samples [2]. More detailed information about such procedures can be found in [2].

To carry out the measurement process, the evaluation method described in [2] was used for the threshold-based algorithm. The measurement procedure for the stepped zones uses three points, each one fitted on the surface of interest using a patch-based strategy. The size of this patches, 1 × 2 mm, allow to cover most of the step surface. In the wedged zones, multiple points were fitted on the surface of interest along three lines of 2 mm length. The point clouds generated by the surface determination algorithms based on a gradient (Canny and Deriche) were processed using Matlab but following the same evaluation method in order to compare both results.

### 2.4. Uncertainty Determination

Expanded measurement uncertainties with level of confidence of 95% (*U*_95_) were calculated according to ISO 14253-2 [16,20] for each one of the measurements carried out over the air wedge gap of the Al/Ti multi-material standard. This standard takes into account the uncertainty contributions related to the CT system length measurement errors with respect to the reference measurements performed with the micro-CMS Zeiss F35, the repeatability of the measurement procedure assessed from five repetitions of the full measurement process (scan, reconstruction, surface determination and measurement) and other uncertainty contributors related to the samples material and temperature stability during the CT measuring process.

## 3. Results

### 3.1. Uncertainty Results Analysis

In order to evaluate the ability of the Canny and Deriche gradient-based algorithms to detect the gap when its width is around voxel size, the uncertainty of CT measurements performed along the wedge air gap of the Al/Ti standard has been determined with the approach explained in Section 2.4.

**Figure 2:**
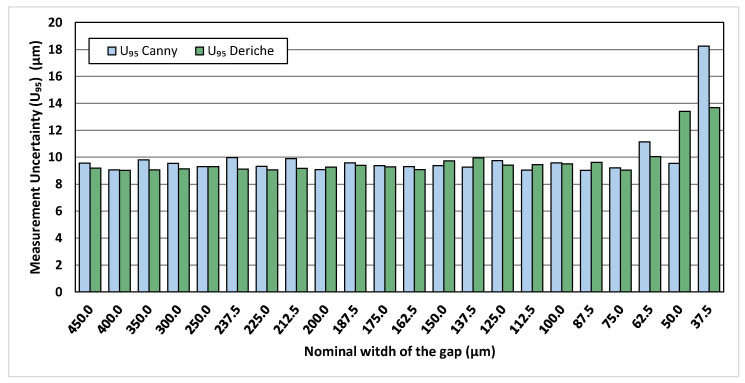
Expanded uncertainty values determined for CT measurements performed along the wedge air gap of the Al/Ti standard using both Canny-based and Deriche-based algorithms.

The measurement uncertainties determined in several positions along the wedge air gap using the Deriche and Canny algorithms are reported in Figure 2. The obtained expanded uncertainties using both algorithms show values below 10 µm for gaps larger than 62.5 µm, and higher values for gaps equal or smaller than 62.5 µm. It is worth noting that this value is around three times the achieved voxel size of 22.1 µm and that, in principle, results might be better in cases when smaller voxel sizes can be obtained, e.g., for samples of smaller dimensions or when scanning smaller portions of the object to be measured. Expanded measurement uncertainty is around 9 µm for these gaps, and, although the Deriche-based algorithm presents better results than the Canny-based one for most tested points, the differences are not very significant. When the gap is narrower than 62.5 µm, the results show that measurement uncertainty increases significantly.

### 3.2. Measurement Error Analysis

**Figure 3:**
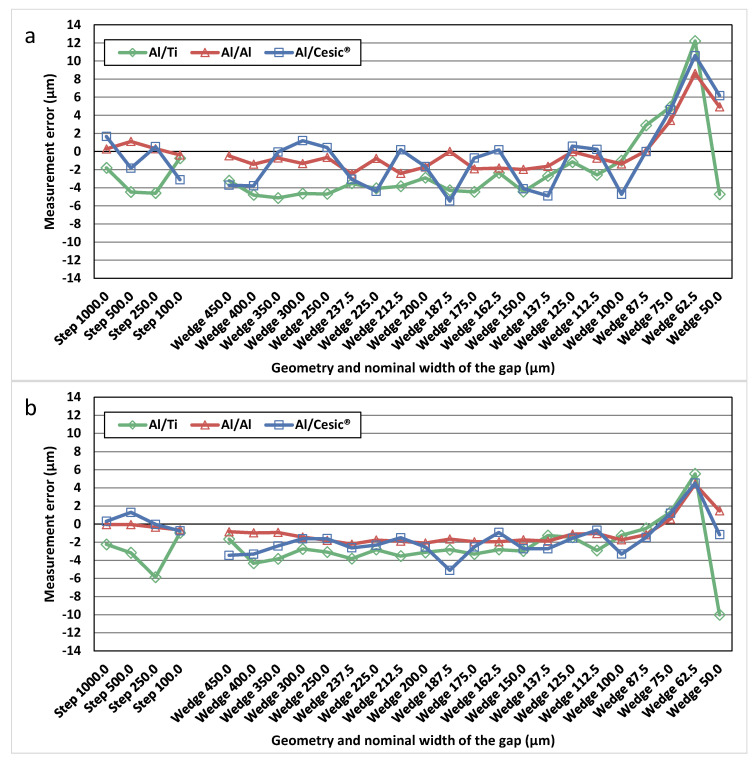
Measurement error using Canny-based algorithm (**a**) and Deriche-based algorithm (**b**) for the three reference standards (Al/Ti, Al/Al and Al/Cesic^®^).

The Canny and Deriche gradient-based algorithms were applied to one CT reconstructed volume of each material combination used in this work to analyse the influence of the materials forming the gap on the performance of the algorithms. In this way, differences in results can be mainly explained by the used surface determination algorithm. Figure 3 shows the measurement errors using the Canny-based and Deriche-based algorithms for each combination of materials. The measurement errors have been calculated as the difference between the measured values and the calibrated values obtained using the CMS Zeiss F25. These measurement errors were calculated in both the stepped and wedged zones of the gap for each standard.

The obtained results show that in all three analysed cases, the measurement error is between 2 and −6 µm except in the areas with a very narrow gap, where the error increases. The measurement error is generally lower for the single-material (Al/Al) standards than for multi-material standards. This effect can be observed with both algorithms, although the differences are slightly smaller when applying the Deriche-based algorithm.

Furthermore, it can be observed that in all the standards used, the measurement errors along the different thicknesses of the gap show lower variability when the Deriche-based algorithm is used. In addition, the measurement error begins to increase and to show a different trend when the thickness of the gap is equal and smaller than 75.0 µm. The obtained expanded uncertainties using both algorithms show values below 10 µm for gaps larger than 62.5 µm, and higher values for gaps equal or smaller than 62.5 µm.

### 3.3. Comparison of Canny and Deriche Gradient-Based Algorithms and Conventional Threshold Algorithms

**Figure 4:**
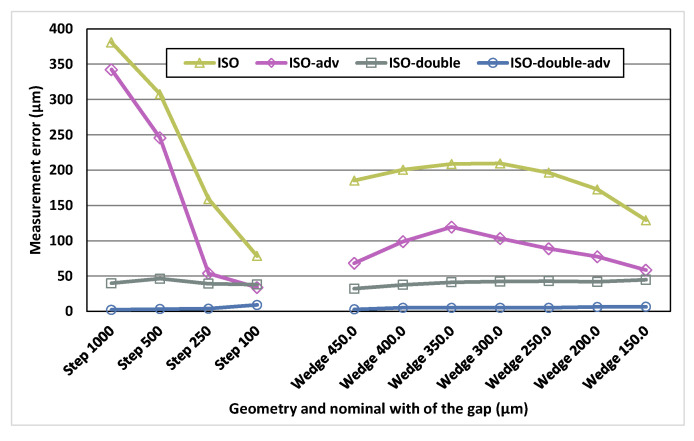
Measurement error results for the Al/Ti multi-material standard of the four threshold-based algorithms provided by VGStudio MAX: ISO, ISO-adv, ISO-double and ISO-double-adv.

Two of the threshold-based surface determination algorithms performed using VGStudio MAX, ISO-double and ISO-doubled-adv, were specifically designed for the multi-material parts in which the absorption coefficients of their materials are clearly different. In these algorithms, two different threshold values were used in order to improve the results, one for the low absorption material and another for the high absorption material. The Al/Ti reference standard is the only one with two clearly different absorption coefficients from those used in this work. Figure 4 shows the measurement errors determined for each of the four threshold-based algorithms available in VGStudio MAX applied to one of the Al/Ti standard.

As can be observed in these results, the lowest measurement errors for the Al/Ti reference standard, especially for large gaps, are achieved when the algorithms with double surface determination are used: ISO-double and ISO-double-adv. For this reason, for the Al/Ti reference standard, only these algorithms will be used in the following to compare the results with those obtained by the Canny and Deriche gradient-based algorithms. Smaller gaps (below 150 µm) were particularly difficult to measure using VGStudio MAX in the case of the Al/Ti multi-material standard, due to an increasing content of noise and image artifacts with respect to mono-material standards. For this reason, only meaningful results obtained with VGStudio MAX are presented and compared with Canny and Deriche gradient-based surface determination algorithms, as shown in the following.

**Figure 5:**
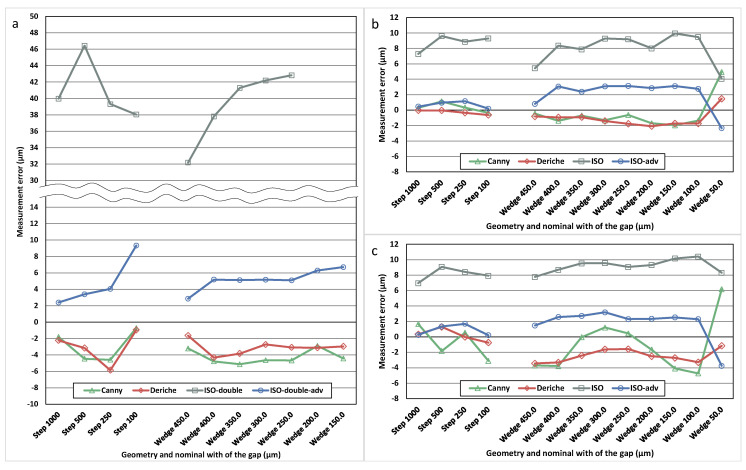
Measurement error results for two surface extraction gradient-based algorithms (Canny and Deriche) and two threshold-based algorithms. The used threshold-based algorithms are ISO-double and ISO-double-adv for the Al/Ti standard (**a**). For Al/Al (**b**) and Al/Cesic^®^ (**c**), the used threshold-based algorithms are ISO and ISO-adv.

In order to evaluate the performance of the Canny and Deriche gradient-based surface determination algorithms, a comparison with commercial conventional threshold-based algorithms has been carried out. Measurement errors achieved using the two gradient-based (Canny and Deriche) and two of the threshold-based algorithms (ISO-double and ISO-double-adv for Ti/Al and ISO and ISO-adv for Al/Al and Al/Cesic^®^ reference standards) in several step and wedged zones of the three reference standards are shown in Figure 5. It is important to note that all the algorithms have been applied to the same reconstructed volumes, so the differences in the results can be mainly assigned to the surface determination algorithm, since the contribution of the previous steps of the measurement procedure is the same for all the algorithms.

Firstly, from the data shown in Figure 5, it can be clearly observed that the ISO 50% based algorithms (ISO and ISO-double), applied to the whole volume, show the worst results, in particular for the Al/Ti reference standard. Another interesting observation is that threshold-based algorithms offer measurement values higher than the reference values, as opposed to Canny and Deriche gradient-based methods, which offer measurement values lower than the reference value.

Analysing the errors obtained with the Canny and Deriche gradient-based algorithms and the best threshold-based algorithm (ISO-double-adv for Al/Ti and ISO-adv for Al/Al and Al/Cesic^®^ reference standards), it can be observed that the differences in the performance of each algorithm are very small.

All these algorithms, when applied to mono-material standards, present increasing measurement errors when the gap goes from 100.0 µm to 50.0 µm (in agreement with what was already observed for Canny-based and Deriche-based algorithms in Figure 3). This is mainly due to the fact that the voxel size of 22.1 µm might be too large to accurately measure gap dimensions smaller than three times the voxel size, as it was already discussed in Section 3.1.

If the sign of the measurement error is not taken into account, focusing on its absolute value, there is not a clear trend in the differences between gradient-based (Canny and Deriche) and ISO-double-adv or ISO-adv surface determination algorithms. With the single-material reference standard (Al/Al) the Canny and Deriche gradient-based algorithms achieve absolute measurement errors around 1 µm lower than ISO-adv, but with the multi-material standard the Deriche-based algorithm does not present differences from ISO-adv (Al/Cesic^®^ reference standard) or ISO-double-adv (Al/Ti reference standard) and the Canny-based algorithm gets only slightly worse results.

There are also no significant differences in the variability of the measurement errors along the gap, although it can be seen that the Deriche-based algorithm has slightly less variability than the threshold-based one, but using the Canny-based algorithm the variability is worse.

## 4. Conclusions

This work was focused on the evaluation of the results obtained using two gradient-based surface determination algorithms, Canny-based and Deriche-based algorithms, in single and multi-material gaps measured by X-ray CT. Such results were also compared with those obtained with the threshold-based algorithms implemented in VGStudio MAX.

When Canny and Deriche gradient-based algorithms are used, the uncertainty determination conducted for the multi-material Al/Ti standard showed that—with the CT scanning parameters used in this work and with a voxel size equal to 22.1 µm—the expanded uncertainty values are below 10 µm for gaps larger than 62.5 µm, while higher values were determined for gaps equal or smaller than 62.5 µm. The measurement errors were found to increase significantly for gaps narrower than 75.0 µm, for all the tested reference standards (Al/Ti, Al/Al, Al/Cesic^®^). Comparing the results obtained by the Canny and Deriche gradient-based algorithms with those achieved by commercial threshold-based algorithms, it was found that the Canny- and Deriche-based algorithms do not lead to larger errors or a further limitation on the minimum measurable gap. However, it should be noted that Canny- and Deriche-based algorithms maintained their measurement capability regardless of the difference in absorptivity of the materials that make up the assembly. In fact, the algorithms included in VGStuido MAX were not able to measure gaps in the wedged area with values below 150 µm in the Al/Ti standard due to the noise present in the image, while the gradient-based algorithms were able to obtain accurate measurements up to gaps of 75 µm.

Analysing the measurement errors achieved using the Canny- and Deriche-based algorithms, it can be seen that the results are worse when the gap is defined between the surfaces of different materials. However, the measurement error increase is only around 3 µm using a Canny-based algorithm and around 1 µm using the Deriche-based algorithm.

It was also observed that the variation in the measurement error along the gap is clearly smaller in the Deriche-based algorithm than in the Canny-based one. Since this aspect had already been observed for other geometries and materials in previous works [14,15], it can be concluded that this better performance of the Deriche-based algorithm is also achieved when measuring difficult-to-measure geometries such as very narrow gaps. Making this same comparison between the Deriche-based and threshold-based algorithms, the differences are not significant, although Deriche might present a slightly lower variability. If error correction techniques are used, this lower variability would simplify the process and improve the accuracy of the corrected measurements.

When using commercial algorithms, it is necessary to choose between simple or double determination depending on the separation between the peaks of the materials observed in the histogram. This choice is not necessary in the studied Canny and Deriche gradient-based algorithms, so a single algorithm is able to get similar results. This is undoubtedly a great advantage for reducing the user influence on the measurement process or even automating it.

## Figures and Tables

**Table 1:** Material properties used in the selected standards.

Material	Composition or Commercial Name	Density (g/cm^3^)	Measured Attenuation Coefficient (200 kV, 1 mm Cu Filter) (mm^−1^)
Aluminium	AlMg4.5Mn0.7	2.46	0.049
Titanium	Ti6Al4V	4.42	0.134
Carbon-fibre reinforced SiC	Cesic^®^	2.65–2.70	0.053

**Table 2:** CT scanning parameters.

Reference Standard	Voltage (KV)	Current (µA)	Exposure Time (MS)	Filter	NO. of Projections	Voxel Size (µM)
Al/Ti	220	50	2898	0.75 mm Cu	2000	22.1
Al/Al	220	50	2000	0.5 mm Cu	2000	22.1
Al/Cesic^®^	220	50	2000	0.5 mm Cu	2000	22.1
